# Comprehensive Transcriptomic Analysis Reveals the Role of the Immune Checkpoint HLA-G Molecule in Cancers

**DOI:** 10.3389/fimmu.2021.614773

**Published:** 2021-07-01

**Authors:** Hui-Hui Xu, Jun Gan, Dan-Ping Xu, Lu Li, Wei-Hua Yan

**Affiliations:** ^1^ Medical Research Center, Taizhou Hospital of Zhejiang Province, Wenzhou Medical University, Linhai, China; ^2^ Key Laboratory of Minimally Invasive Techniques & Rapid Rehabilitation of Digestive System Tumor of Zhejiang Province, Taizhou Hospital of Zhejiang Province, Linhai, China; ^3^ Reproductive Center, Taizhou Hospital of Zhejiang Province, Wenzhou Medical University, Linhai, China; ^4^ Pediatrics, Taizhou Hospital of Zhejiang Province, Wenzhou Medical University, Linhai, China

**Keywords:** HLA-G, immune checkpoint, RNAseq, bioinformatics analysis, survival, tumor-infiltrating immune cells

## Abstract

Human leukocyte antigen G (HLA-G) is known as a novel immune checkpoint molecule in cancer; thus, HLA-G and its receptors might be targets for immune checkpoint blockade in cancer immunotherapy. The aim of this study was to systematically identify the roles of checkpoint HLA-G molecules across various types of cancer. ONCOMINE, GEPIA, CCLE, TRRUST, HAP, PrognoScan, Kaplan-Meier Plotter, cBioPortal, LinkedOmics, STRING, GeneMANIA, DAVID, TIMER, and CIBERSORT were utilized. Gene Ontology (GO) and Kyoto Encyclopedia of Genes and Genomes (KEGG) enrichment analyses were performed. In this study, we comprehensively analysed the heterogeneous expression of HLA-G molecules in various types of cancer and focused on genetic alterations, coexpression patterns, gene interaction networks, HLA-G interactors, and the relationships between HLA-G and pathological stage, prognosis, and tumor-infiltrating immune cells. We first identified that the mRNA expression levels of HLA-G were significantly upregulated in both most tumor tissues and tumor cell lines on the basis of in-depth analysis of RNAseq data. The expression levels of HLA-G were positively associated with those of the other immune checkpoints PD-1 and CTLA-4. Abnormal expression of HLA-G was significantly correlated with the pathological stage of some but not all tumor types. There was a significant difference between the high and low HLA-G expression groups in terms of overall survival (OS) or disease-free survival (DFS). The results showed that HLA-G highly expressed have positive associations with tumor-infiltrating immune cells in the microenvironment in most types of tumors (*P*<0.05). Additionally, we identified the key transcription factor (TF) targets in the regulation of HLA-G expression, including HIVEP2, MYCN, CIITA, MYC, and IRF1. Multiple mutations (missense, truncating, etc.) and the methylation status of the *HLA-G* gene may explain the differential expression of HLA-G across different tumors. Functional enrichment analysis showed that HLA-G was primarily related to T cell activation, T cell regulation, and lymphocyte-mediated immunity. The data may provide novel insights for blockade of the HLA-G/ILT axis, which holds potential for the development of more effective antitumour treatments.

## Introduction

Human leukocyte antigen G (HLA-G), a tolerogenic non-classical HLA molecule, plays an important role in modulating the response of maternal immune cells that contribute to the maintenance of tolerance of the semi-allogenic fetus during pregnancy (1). HLA-G has a highly tissue-restricted expression pattern on physiological tissues, such as the placenta, pituitary gland and testis (Supporting Information **Supplementary Figure 1**). However, under pathological conditions, HLA-G has been detected in many types of primary tumors and metastases at a variable frequency and is strongly related to high tumor grade and poor prognosis for cancer patients (2–4). The immunosuppressive properties of HLA-G can also be exploited by tumors to escape recognition by the host immune system. Its highly tissue-specific physiological expression profile and immunosuppressive functions have made HLA-G an interesting research topic in recent decades, especially for cancer research. Based on the fact that HLA-G is a normal immune signal capable of stopping an immune response, many studies have claimed that HLA-G might be a potentially novel immune checkpoint in tumors that functions in combination with other immune checkpoints, such as programmed cell death protein 1 (PD-1) and cytotoxic T lymphocyte-associated protein 4 (CTLA-4) (5–10).

Functionally, HLA-G has broad immunosuppressive properties that affect both innate and adaptive immune responses. HLA-G functions in multiple steps to weaken antitumour immune responses by acting on through its receptors present on immune cells, including ILT2 (LILRB1/CD85j), ILT4 (LILRB2/CD85d), and KIR2DL4 (CD158d) (11, 12). HLA-G inhibits the cytolytic function of uterine and peripheral blood NK cells, cytotoxic T lymphocyte (CTL)-mediated cytolysis, alloproliferative response of CD4+ T cells, proliferation of T cells and peripheral blood NK cells, and maturation and function of dendritic cell (DCs) or B cells. HLA-G also induces regulatory T cells (Tregs) and myeloid suppressor cells (MSCs) (13–15). In contrast to both PD-1 and CTLA-4, HLA-G is capable of blocking all stages of the antitumour response, from APC activation and effector priming to the functions of fully activated CTLs, NK cells and B cells (16). Thus, HLA-G and its receptors might be targets for immune checkpoint blockade to restore immune cell function during cancer immunotherapy but still require further investigation. The aim of this study was to identify the roles of HLA-G molecules across various types of cancer.

To determine whether HLA-G serves as an immune checkpoint molecule in cancer, we conducted an in-depth and comprehensive bioinformatics analysis of the expression of HLA-G in various types of cancers and focused on genetic alterations, coexpression patterns, gene interaction networks, and HLA-G interactors, as well as the relationships between HLA-G and pathological stage, prognosis, and tumour-infiltrating immune cells.

## Materials and Methods

### ONCOMINE

ONCOMINE (https://oncomine.org) is a cancer gene data-mining platform that contains 715 datasets and 86,733 samples and enables genome-wide expression and DNA copy number analysis (17). The data analysis included only mRNA subtypes in this study. We evaluated the tumor expression patterns of HLA-G, its receptors, and other immune checkpoint molecules in different types of tumors. A simultaneous fold change of 1.5, gene rank in the top 10% and *P*-value of 0.05 was set as the threshold. Student’s t test was used to analyze the tumor *vs.* normal differential expression of HLA-G.

### GEPIA

Gene Expression Profiling Interactive Analysis (GEPIA, http://gepia.cancer-pku.cn/index.html) is an interactive web server for analyzing the RNA sequencing (RNAseq) expression data of 9,736 tumors and 8,587 normal tissues from The Cancer Genome Atlas (TCGA) and Genotype-Tissue Expression (GTEx) projects using a standard processing pipeline (18). In this study, we determined the correlation between HLA-G expression and pathological stage, overall survival (OS), and disease-free survival (DFS, also called relapse-free survival, RFS) with the “Single Gene Analysis” module of GEPIA. Pathological stage was analysed by ANOVA. The survival analysis employed the Cox proportional hazard model to calculate the hazards ratio (HR).

### CCLE

The Cancer Cell Line Encyclopedia (CCLE, https://portals.broadinstitute.org/ccle) project contains 1457 cell lines that provide detailed genetic and pharmacologic characterization of a large panel of human cancer models and enable users to translate cell line integrative genomics data into cancer patient stratification strategies (19). In this study, we analysed the expression and methylation level of HLA-G in each tumor cell line using the search term “HLA-G”.

### TRRUST

Transcriptional Regulatory Relationships Unraveled by Sentence-based Text mining (TRRUST, https://grnpedia.org/trrust/) is a manually curated database of human and mouse transcriptional regulatory networks (20). Currently, TRRUST contains 8,427 transcription factor (TF)-target regulatory relationships of 795 human TFs and provides information on how these interactions are regulated.

### HPA

The Human Protein Atlas (HPA, https://proteinatlas.org/) aims to map all human proteins in cells, tissues and organs using the integration of various omics technologies, including antibody-based imaging, mass spectrometry-based proteomics, transcriptomics and systems biology (21). HPA consists of six separate parts: Tissue Atlas, Cell Atlas, Pathology Atlas, Blood Atlas, Brain Atlas, and Metabolic Atlas. In this study, we analysed the protein expression of HLA-G in various types of human physiological tissues and/or cells.

### PrognoScan

PrognoScan (http://www.prognoscan.org/) is a new database for meta-analysis of the prognostic value of genes that provides a powerful platform for evaluating potential tumor markers and therapeutic targets (22). We evaluated the prognostic values of HLA-G in the various types of tumors, and overall survival (OS) and relapse-free survival (RFS) were included in this study. *P*-values <0.05 were indicated a significant difference.

### Kaplan-Meier Plotter

The Kaplan-Meier Plotter (http://kmplot.com/analysis/) database is capable of assessing the effect of 54,000 genes (mRNA, miRNA, and protein) on survival in 21 cancer types, including breast (n=6,234), ovarian (n=2,190), lung (n=3,452), and gastric (n=1,440) cancer (23). Sources for the databases include Gene Expression Omnibus (GEO), EGA, and TCGA. The primary purpose of the tool is the meta-analysis-based discovery and validation of survival biomarkers. We evaluated the associations between HLA-G expression and cancer survival. In this study, we split the patients according to the automatically selected HLA-G expression cutoff, and a follow-up of 240 months was set as the threshold.

### cBioPortal

The cBioPortal for Cancer Genomics (http://cbioportal.org) platform provides a comprehensive web resource of cancer genomics data from multiple platforms for exploration, network visualization, and survival analysis (24). In this study, genetic alterations, coexpression patterns, and the interaction network of HLA-G were obtained from cBioPortal. HLA-G mRNA coexpression z scores (RNA Seq V2 RSEM) were obtained using a z score threshold of ± 2.0.

### LinkedOmics

LinkedOmics (http://linkedomics.org) is a unique, publicly available platform for biologists and clinicians to access, analyze and compare cancer multiomics data within and across tumor types that contains multiomics data from all 33 TCGA cancer types (25). In this study, we explored the potential coexpression genes of HLA-G. The Pearson correlation test was employed for statistical analysis.

### STRING

STRING (http://string-db.org) is a database of known and predicted protein-protein interactions (PPIs) (26). The interactions include direct (physical) and indirect (functional) associations; they stem from computational prediction, from knowledge transfer between organisms, and from interactions aggregated from other (primary) databases. The STRING database currently covers 24,584,628 proteins from 5,090 organisms. In this study, we performed a PPI network analysis to explore HLA-G *via* STRING.

### GeneMANIA

GeneMANIA (http://genemania.org) is a flexible, user-friendly website that provides information for genetic interactions and gene functional assays (27). To date, hundreds of datasets and hundreds of millions of interactions have been collected from GEO, BioGRID, IRefIndex and I2D, as well as organism-specific functional genomics datasets. We explored the interactions between HLA-G and its neighboring genes in this study.

### DAVID

The Database for Annotation, Visualization and Integrated Discovery V6.8 (DAVID, https://david.ncifcrf.gov/) provides a comprehensive set of functional annotation tools for investigators to understand the biological meaning behind a large list of genes (28). In this study, Gene Ontology (GO) enrichment analysis and Kyoto Encyclopedia of Genes and Genomes (KEGG) pathway enrichment analysis of HLA-G and its coexpression genes were performed *via* DAVID, and data were visualized with the “clusterProfiler” R package for molecules of interest with *P*-values < 0.05. Biological process (BP), cellular component (CC), and molecular function (MF) terms were derived from the GO enrichment analysis.

### TIMER

The Tumor Immune Estimation Resource (TIMER, https://cistrome.shinyapps.io/timer/) is a comprehensive resource for the systematic analysis of immune infiltrates across diverse cancer types from the TCGA database and includes 10,897 samples of 32 cancer types (29). The abundances of six immune infiltrates (B cells, CD4+ T cells, CD8+ T cells, neutrophils, macrophages, and dendritic cells) were estimated to assess tumor immunological, clinical, and genomic features. In this study, “Gene module” was used to evaluate the correlation between the expression level of HLA-G and the typical infiltration of immune cells. The scatterplots of HLA-G and different immune infiltrates are displayed, showing the purity-corrected partial Spearman’s correlation and statistical significance.

### CIBERSORT

CIBERSORT (https://cibersort.stanford.edu/) is a gene expression-based deconvolution algorithm that provides an estimation of the abundances of member cell types in a mixed cell population using gene expression data (30). In this study, we estimated the correlation between the expression level of HLA-G and the relative proportions of 22 distinct functional subsets of immune cells.

## Results

### Expression of the Immune Checkpoint HLA-G in Tumors

**Figure 1:**
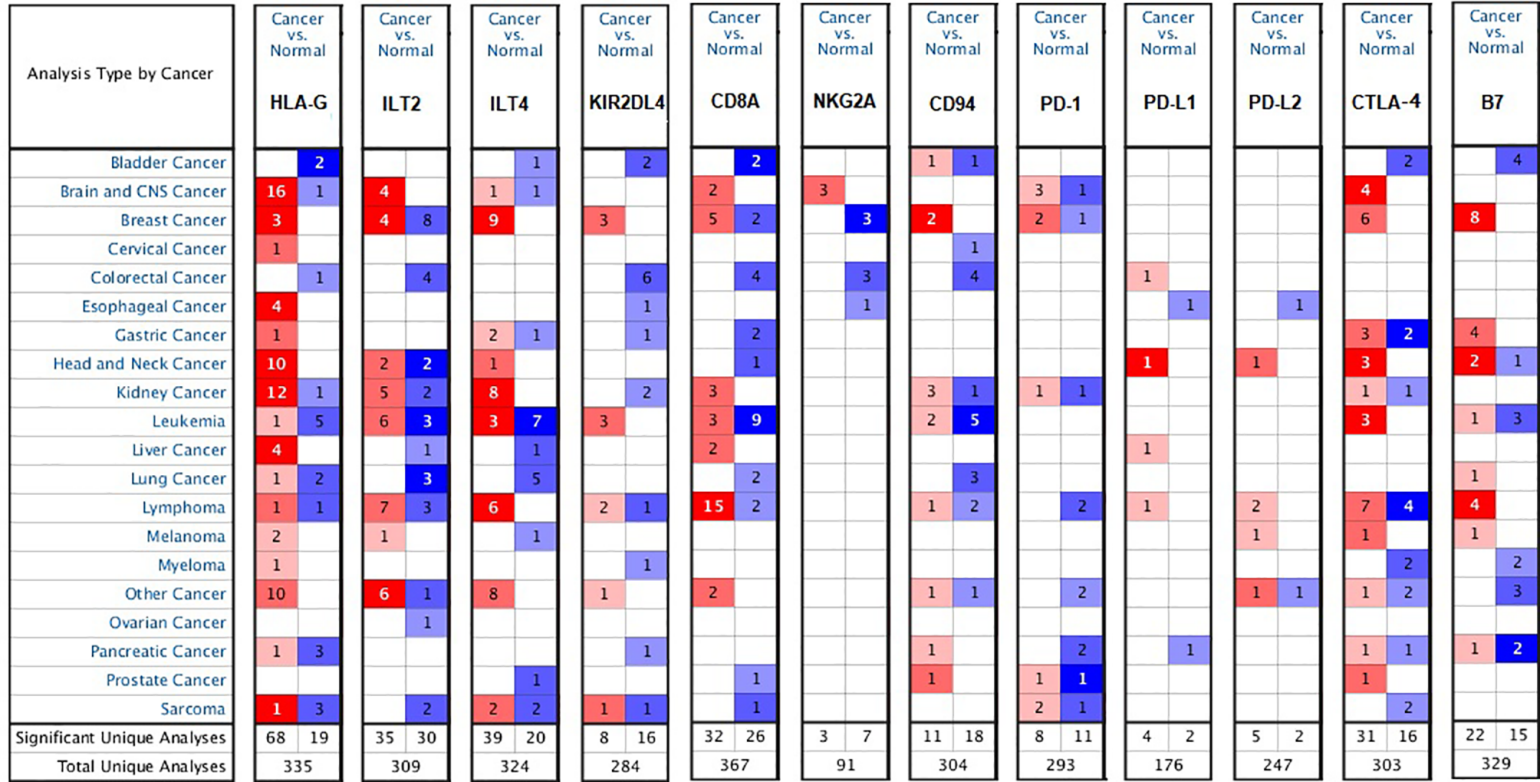
Differential expression of the checkpoint molecules HLA-G, PD-1, CTLA-4 and their receptors in the various types of human tumors (ONCOMINE database). Graph shows the number of datasets with a statistically mRNA overexpression, based on the cut-off value of *P* < 0.05, fold change >1.5, and gene rank in the top 10% in the ONCOMINE database. Red represents over-expression, blue represents down-expression, and white represents no difference in tumors *vs.* normal adjacent tissues.

We investigated the mRNA levels of the checkpoints HLA-G, PD-1 (PDCD1), and CTLA-4 and their receptors in various types of human tumors and/or adjacent normal tissues using the ONCOMINE database (**Figure 1**). Based on the datasets from the ONCOMINE platform, the mRNA levels of HLA-G were significantly elevated in 78.2% tumors (68 analyses showing higher expression *vs.* 19 analyses showing lower expression), especially in testicular embryonal carcinoma (fold change=8.262 and *P*=0.003), clear cell renal cell carcinoma (fold change=8.072 and *P*=6.44E-5), melanoma (fold change=7.974 and *P*=3.31E-5), pancreatic adenocarcinoma (fold change=6.739 and *P*=0.002), and glioblastoma (fold change=6.592 and *P*=8.80E-18) (Supporting Information **Supplementary Table 1**) (31–67).

**Figure 2:**
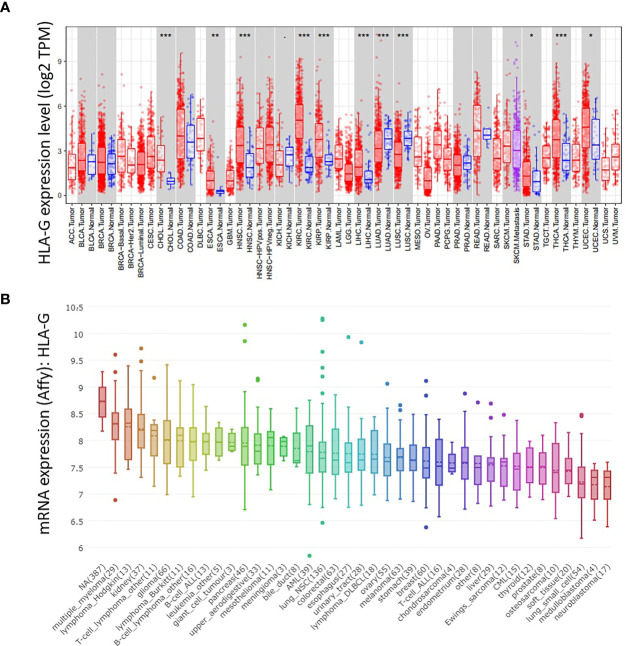
The mRNA expression level of HLA-G in the various types of human tumors. **(A)** DiffExp module showing the differential expression between tumor and adjacent normal tissues for HLA-G mRNA across all TCGA tumors in the TIMER database. Up- or down-regulated in the tumors compared to normal tissues for each cancer type, as displayed in gray columns when normal data are available. *P*-value Significant Codes: 0 ≤ *** < 0.001 ≤ ** < 0.01 ≤ * < 0.05 ≤. < 0.1. **(B)** The mRNA expression of HLA-G in different cancer cell lines using Affy gene chip data in the CCLE database.

To verify the validity of the above results from ONCOMINE database, we used other databases (TIMER and CCLE) to analyze the expression of HLA-G in various types of human tumors. As depicted in **Figure 2A**, HLA-G expression was also significantly upregulated in most tumors, especially in kidney renal clear cell carcinoma (KIRC) (*P*=3.39E-32), head and neck squamous cell carcinoma (HNSC) (*P*=1.58E-08), thyroid carcinoma (THCA) (*P*=2.66E-07), kidney renal papillary cell carcinoma (KIRP) (*P*=4.21E-06), liver hepatocellular carcinoma (LIHC) (*P*=1.16E-06), cholangiocarcinoma (CHOL) (*P*=1.26E-05), esophageal carcinoma (ESCA) (*P*=0.001), uterine corpus endometrial carcinoma (UCEC) (*P*=0.014), and stomach adenocarcinoma (STAD) (*P*=0.035) but downregulated in lung adenocarcinoma (LUAD) (*P*=0.0009) and lung squamous cell carcinoma (LUSC) (*P*=8.29E-11). In addition, analysis from the CCLE database was consistent with these results, confirming that HLA-G is ectopic expressed in most human tumor cells (**Figure 2B**). Thus, HLA-G molecule exhibits highly variable expression among tumor types, with marked intertumoral heterogeneity.

**Figure 3:**
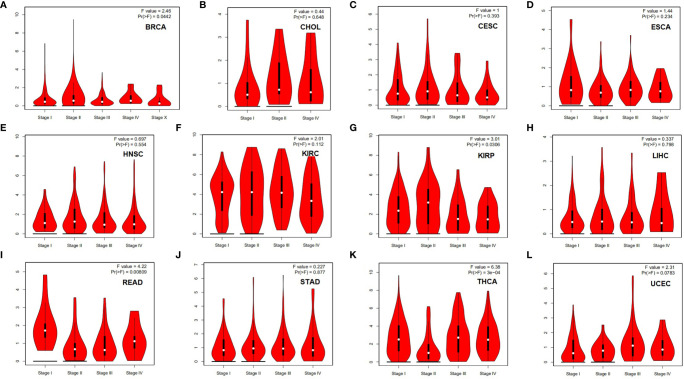
Correlation between HLA-G expression and the pathological stage in different types of human tumors (GEPIA). *P*-value < 0.05. **(A)** BRCA, **(B)** CESC, **(C)** CHOL, **(D)** ESCA, **(E)** HNSC, **(F)** KIRC, **(G)** KIRP, **(H)** LIHC, **(I)** READ, **(J)** STAD, **(K)** THCA, **(L)** UCEC.

To evaluate the role of HLA-G in tumor progression, we assessed the correlation between HLA-G expression and pathological stage in different tumor patients using the GEPIA database. HLA-G expression was significantly associated with the pathological stage of breast invasive carcinoma (BRCA) (*P*=0.044), KIRP (*P*=0.031), rectal adenocarcinoma (READ) (*P*=0.008), and THCA (*P*=3E-04) (**Figure 3**).

### Prognostic Value of HLA-G in Human Tumors

**Figure 4:**
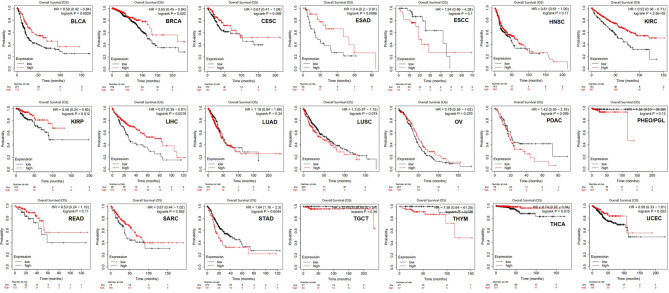
The prognostic values of HLA-G expression on overall survival in 21 cancer types patients (Kaplan-Meier Plotter). Survival curves for high (red) and low (black) expression groups dichotomized at the optimal cutpoint are plotted. The X-axis represents time and the Y-axis represents survival rate.

The correlations between HLA-G and clinical outcomes in the various types of tumors were explored using the Kaplan-Meier Plotter database. The data suggested that HLA-G expression was significantly favorably associated with overall survival (OS) in bladder urothelial carcinoma (BLCA) (*P*=0.0029), BRCA (*P*=0.022), ESAD (*P*=0.0086), KIRC (*P*=2.6E-05), KIRP (*P*=0.012), LIHC (*P*=0.0019), and THCA (*P*=0.015). In addition, higher mRNA levels of HLA-G in STAD (*P*=0.0044) and thymoma (THYM) (*P*=0.0026) were significantly associated with shorter OS (**Figure 4**). Relapse-free survival (RFS, also called disease-free survival, DFS) curves were generated and are presented in Supporting Information **Supplementary Figure 2**. The results showed that patients with higher HLA-G expression in the BLCA (*P*=3.4E-05), KIRC (*P*=0.029), KIRP (*P*=0.021), LIHC (*P*=0.014), and LUAD (*P*=0.039) groups were had significantly longer RFS, whereas higher HLA-G in the pancreatic ductal adenocarcinoma (PDAC) (*P*=0.011) group was significantly associated with shorter RFS.

Additionally, we exploited the RNAseq data in TCGA using the GEPIA platform to explore the prognostic value of HLA-G. As shown in Supporting Information **Supplementary Figure 3A**, HLA-G expression had a significant favorable association with OS in KIRC (*P*=0.027), MESO (*P*=0.041), and THCA (*P*=0.019). The data suggest that GBM (*P*=0.0084) patients with high HLA-G expression were significantly more likely to have a shorter disease-free survival (DFS) than patients with GBM with low HLA-G expression (Supporting Information **Supplementary Figure 3B**). The data suggest that high expression of HLA-G had significant prognostic value in KIRC and THCA in analyses using both the Kaplan-Meier Plotter and GEPIA databases.

### *HLA-G* Methylation, Mutation, and HLA-G Transcription Factor Targets in Tumors

**Figure 5:**
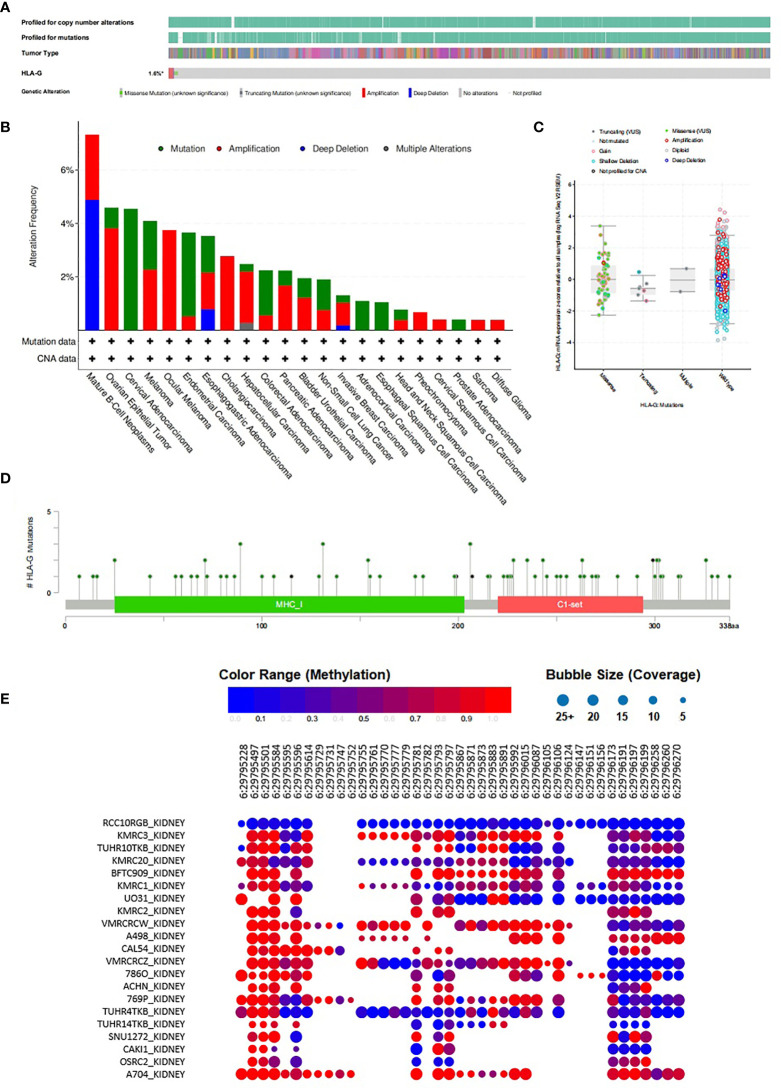
Methylation and mutation status of *HLA-G* gene in tumors. **(A)**Summary of genetic alterations in *HLA-G* in tumors (cBioPortal), **(B)** The distribution of these *HLA-G* genetic variants in different types of tumors, **(C)** HLA-G expression analysis and mutation status in human tumors using cBioPortal, **(D)** Mutation status of *HLA-G* in human tumors from cBioPortal, **(E)** Methylation status of HLA-G in kidney cancer cell lines from CCLE database.

To further understand the intertumoral heterogeneity of HLA-G expression among different tumors, we performed comprehensive analyses of the characteristics of the *HLA-G* gene using the cBioPortal and CCLE databases. As depicted in **Figure 5A**, the rate of *HLA-G* gene alteration was 1.6% in 10,953 patients and 10,967 tumor samples from cBioPortal. The distribution of these *HLA-G* genetic variants in different types of tumors is shown in **Figure 5B**. Using the reference sequence NM_002127, multiple mutations in *HLA-G* have been identified in human tumors, such as missense mutations, truncating mutations, amplifications, and deep deletions. Data showed that the *HLA-G* gene contains 81 mutations, including 72 missense and 9 truncating mutations. Missense mutations were the major form of mutation (**Figures 5C** **, D**).

The bubble chart showed the methylation level of *HLA-G* in kidney cancer cell lines data from the CCLE database (**Figure 5E**). HLA-G was highly methylated at three sites (positions 29,795,497, 29,795,501 and 29,795,584) on chromosome 6. The discovery of CpG island methylation further supported the differential expression pattern of HLA-G in different types of human tumors.

In addition, we explored possible transcription factor targets of HLA-G using the TRRUST database. HLA-A, HLA-B, HLA-C, HLA-E, HLA-F, HLA-G, B2M, LILRB1, LILRB2, KIR2DL4, CD8A, CD160, TAP1, and TAP2 data are included in TRRUST. As shown in **Table 1**, our analysis suggest that five transcription factor targets (HIVEP2, MYCN, CIITA, MYC, and IRF1) are associated with the regulation of HLA-G expression.

**Table 1:** Key regulated factor of HLA I and their receptors in tumor (TRRUST).

Key TF	Description	Regulated gene	P value	FDR
HIVEP2	human immunodeficiency virus type I enhancer binding protein 2	HLA-A, HLA-B, HLA-C, HLA-E, HLA-F, HLA-G	1.28E-19	1.15E-18
MYCN	v-myc myelocytomatosis viral related oncogene, neuroblastoma derived (avian)	HLA-A, HLA-B, HLA-C, HLA-E, HLA-F, HLA-G	1.02E-12	4.59E-12
CIITA	class II, major histocompatibility complex, transactivator	HLA-A, HLA-B, HLA-C, HLA-F, HLA-G	3.67E-11	1.10E-10
MYC	v-myc myelocytomatosis viral oncogene homolog (avian)	HLA-A, HLA-B, HLA-C, HLA-E, HLA-F, HLA-G	1.46E-10	3.28E-10
IRF1	interferon regulatory factor 1	HLA-A, HLA-G,TAP1, TAP2	8.40E-08	1.51E-07
TRERF1	transcriptional regulating factor 1	LILRB1, LILRB2	1.41E-05	2.12E-05

TF, transcription factor.

### Coexpression Patterns, Gene Interaction Networks, and HLA-G Interactors

**Figure 6:**
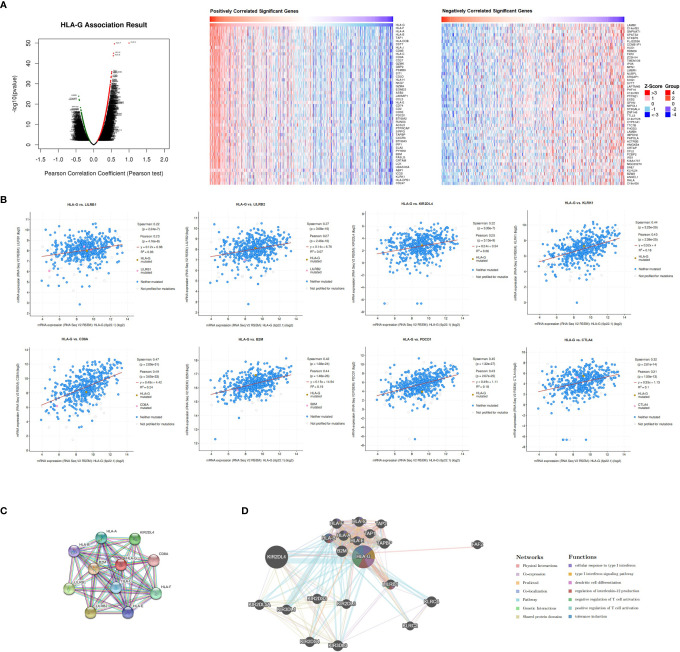
Co-expression, neighbor gene network, and interaction analysis of HLA-G. **(A)** Co-expression and correlation heat map of HLA-G in KIRC, **(B)** Correlation between HLA-G expression and its receptors, **(C, D)** Protein-protein interaction network of HLA-G.

Due to the high transcriptional levels of HLA-G having significant prognostic value in KIRC, we decided to explore molecules coexpressed with HLA-G in KIRC using the LinkedOmics and cBioPortal databases. As shown in Supporting Information **Supplementary Table 2** and **Figure 6A**, the top 50 positively correlated, significant coexpressed genes were HLA-F, HLA-B, HLA-A, HLA-H, HLA-C, HLA-J, TAP1, CST7, CD27, HLA-DOB, GZMK, CD8B, CD8A, GBP2, SIRPG, JAKMIP1, RUNX3, PSMB9, SIT1, NKG7, GZMA, CD3D, CD3E, CXCR3, CCL5, CD2, PDCD1, IRF1, ASB2, ACSL5, CD74, PTPRCAP, EOMES, PYHIN1, CRTAM, PSMB10, KLRK1, BTN3A2, IFNG, CORO1A, FASLG, LAMB1, LCK, UBASH3A, SLA2, EHD1, IL2RG, ZBED2, ICOS, and B2M. The mRNA expression levels of HLA-G were positively associated with the expression of its receptors (LILRB1, LILRB2, and KIR2DL4) and other immune checkpoint molecules (PD-1 and CTLA-4) (**Figure 6B**).

Furthermore, we conducted a PPI network analysis of HLA-G with STRING to explore its potential interactions with other molecules. As a result, 11 nodes and 49 edges were obtained in the PPI work (PPI enrichment *P*-value <1.0E-16) (**Figure 6C**). The results of the GeneMANIA analysis revealed that the function of HLA-G was primarily related to dendritic cell differentiation, regulation of interleukin-12 production, positive regulation of T cell activation, and tolerance induction (**Figure 6D**).

### Functional Enrichment Analysis of HLA-G

**Figure 7:**
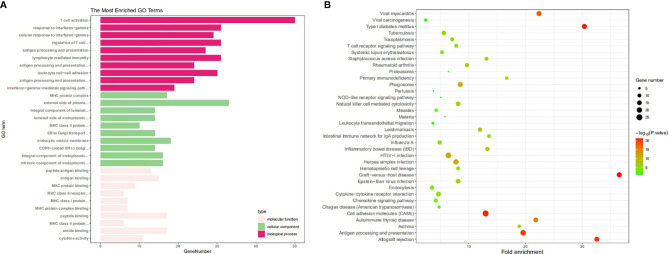
The enrichment analysis of HLA-G and 50 most frequently altered neighboring genes (DAVID). **(A)** Bar plot of GO enrichment in cellular component terms, biological process terms, and molecular terms, **(B)** Kyoto Encyclopedia of Genes and Genomes (KEGG) pathway analysis.

We then explored the functions of HLA-G and its coexpression genes (the top 2000 genes in **Supplementary Table 2**) using DAVID and R software. As depicted in **Figure 7A**, among the 10 most highly enriched GO functions in the biological process (BP) category, T cell activation, lymphocyte differentiation, regulation of T cell activation, leukocyte cell-cell adhesion, and T cell differentiation were the pathways identified associated with tumorigenesis and progression. In the cellular component (CC) category, external side of plasma membrane, endocytic vesicle, phagocytic vesicle, MHC protein complex, and integral component of lumen were the main GO terms. In the molecular function (MF) category, HLA-G and its coexpression genes were mainly enriched in immune receptor activity, peptide antigen binding, MHC protein complex binding, chemokine activity, and tumor necrosis factor receptor binding.

Kyoto Encyclopedia of Genes and Genomes (KEGG) pathway analysis was also performed. Data showed that among the top 10 KEGG pathways, Epstein-Barr virus infection, cell adhesion molecule (CAM), NK cell mediated cytotoxicity, Th17 cell differentiation, and antigen processing and presentation were significantly associated with tumorigenesis and progression (**Figure 7B**).

### Relationship Between HLA-G and Immune Infiltration in the Tumor Microenvironment

Although the data showed that upregulated expression of HLA-G in tumor tissues impacted the prognosis of human tumors, the underlying mechanisms were still unclear. Therefore, we embarked on a comprehensive exploration of the correlation between HLA-G expression and immune cell infiltration using the TIMER database. As shown in Supporting Information **Supplementary Figure 4**, the expression of HLA-G was significantly associated with the infiltration of B cells, CD4+ T cells, CD8+ T cells, neutrophils, macrophages and dendritic cells in 33 different types of tumors (*P* < 0.05). There was a positive correlation between HLA-G expression and the infiltration of B cells (Cor=0.338, *P*=9.50E-14), CD4+ T cells (Cor=0.123, *P*=8.14E-03), CD8+ T cells (Cor=0.348, *P*=8.35E-14), neutrophils (Cor=0.242, *P*=1.57E-07), macrophages (Cor=0.128, *P*=6.66E-03) and dendritic cells (Cor=0.28, *P*=1.28E-09) in KIRC using TIMER.

**Figure 8:**
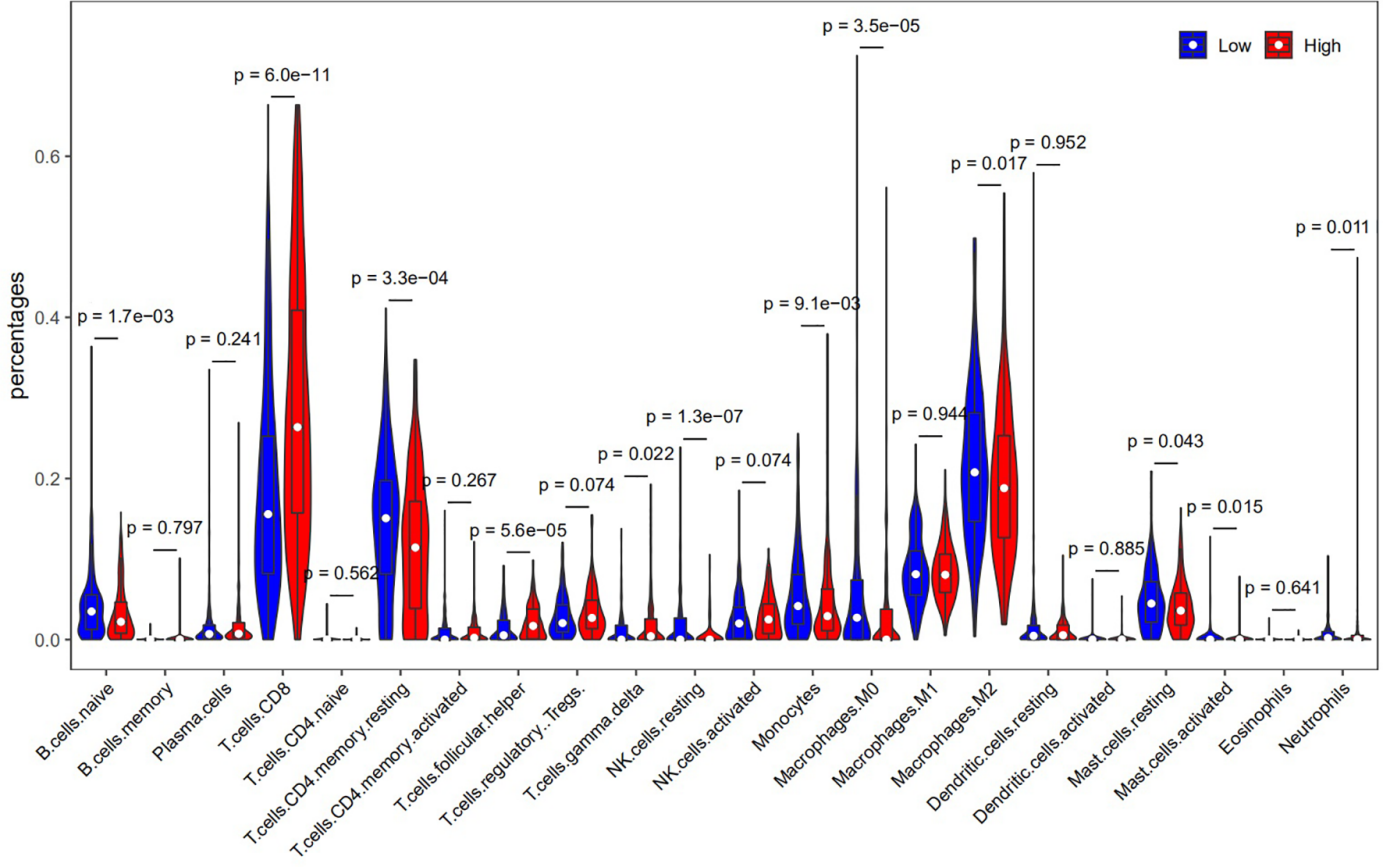
The correlation between HLA-G expression and immune cell infiltration (CIBERSORT).

We also confirmed the association between HLA-G expression and immune subsets using CIBERSORT (Supporting Information **Supplementary Table 3**). As depicted in **Figure 8**, there was a positive correlation between HLA-G expression and the infiltration of CD8+ T cells (*P*=6.0E-11). The number of Tregs (which function as immune suppressors) might be associated with the expression of HLA-G (*P*=0.074).

## Discussion

HLA-G is a potentially novel immune checkpoint molecule in cancer, but additional evidence is required to provide. In recent decades, accumulating evidence has shown that HLA-G is a tumor biomarker that plays key roles in tumorigenesis, tumor cell proliferation, invasion and metastasis (2–4, 8). Indeed, HLA-G is capable of inhibiting all stages of the antitumour response *via* its inhibitory receptors ILT2 and ILT4. Previously, our *in vitro* studies provided that HLA-G expression in hepatocellular carcinoma and ovarian carcinoma cell lines could directly inhibit NK cell lysis, and blocking with HLA-G antibodies restored the NK-mediated lysis of the targeted cancer cell lines (68, 69). Our *in vivo* study provided that blocking HLA-G with a specific neutralizing antibody can inhibit the growth of HLA-G-positive tumor cells and restore antitumour immunity against HLA-G-positive tumor cells in a humanized mouse model of ovarian cancer (70). In humans, we have widely studied the relationship between HLA-G expression and clinical outcomes in different tumor types, including solid tumors (3, 4, 68–77) and hematologic malignancies (78, 79). Our previous studies also explored the regulation mechanisms of HLA-G expression and function (80–82). In addition to HLA-G expressed on tumor cells, intercellular transfer of tumor cell-derived HLA-G molecules through trogocytosis, exosomes and tunnelling nanotubes (TnTs), which represents another important complementary mechanism for cancer cell escape (83). Based on these results, we agree that HLA-G might be a novel immune checkpoint in cancer. Importantly, immune checkpoint inhibitors (ICIs) are an evolving treatment option for several types of cancer, but only a limited number of patients benefit from such therapy. For example, antibody to PD-1 (nivolumab) has made some progress in patients with NSCLC; however, most tumors are still unresponsive (84). Several ongoing clinical trails are investigating the combination therapy of ICIs, and it is urgent to establish novel complementary biomarkers. Because HLA-G and PD-L1 have distinct but overlapping expression patterns, Tizona’s HLA-G antagonist may help patients who do not respond to current anti-PD-1/PD-L1 treatments and deepen responses among those sensitive to existing immunotherapy agents (85). Therefore, understanding the relationship between HLA-G-positive tumors and its microenvironment is critical to developing effective immunotherapeutic strategies.

In this study, we first systematically identified that the expression of HLA-G in various types of cancers. Based on different databases, the bioinformatics analysis showed that HLA-G was upregulated in most types of human tumor tissues or tumor cell lines (**Figure 2**). The results were consistent with previous studies that the intertumoral and intratumoral heterogeneity of HLA-G expression was commonly seen across different tumor types (3, 4, 8). HLA-G can inhibit the activities of all immune cells by interacting with its receptors ILT2 and ILT4, thus protecting tumor cells from host antitumour responses (9–11, 13). HLA-G is a good target, based upon this specificity and its activity on about most human tumors. In addition, our analysis showed that the expression levels of HLA-G were positively correlated with those of its receptors ILT2 and ILT4 and the other immune checkpoints PD-1 and CTLA-4 (**Figure 6**). Consistent with the present results, the findings of a previous study revealed coexpression of HLA-G and ILT4 in primary colorectal cancer (CRC) and identified its association with advanced stage and poor OS (86). Other studies showed abnormally high expression of HLA-G in clear cell renal cell carcinoma, potentially in association with high PD-1 (9, 87). Interestingly, we found that PD-1 was one of the top 50 positively correlated significantly coexpressed genes of HLA-G; however, studies on the interaction between these checkpoints are rare. Therefore, further illumination of the relationship between HLA-G and other immune checkpoints (PD-1, CTLA-4, etc.) is meaningful for better selection of immunotherapeutic targets and application in combination therapy of ICIs.

We further identified correlations between HLA-G expression and disease stage or clinical outcomes in patients with different types of tumors. In the present study, the expression of HLA-G was correlated with the pathological stages in some tumors, such as BRCA, KIRP, READ, and THCA (**Figure 3**). This result confirmed the immunosuppressive function of HLA-G in the tumorigenesis and progression of these tumors. Consistent with previous studies, HLA-G expression was associated with the developmental stage of cervical cancer and non-small-cell lung cancer (NSCLC) patients (77, 88). However, it was also reported that there was no association between HLA-G expression and the disease stage in thyroid tumors (89). The prognostic values of HLA-G varied greatly across cancers. We observed a significant difference between the high and low HLA-G expression groups in overall survival (OS) or disease-free survival (DFS) in some but not all tumor types (**Figure 4**). Our study was the first to identify HLA-G as a predictor of good prognosis for KIRC and THCA at transcriptional level through the Kaplan-Meier Plotter and GEPIA databases. Considering the immunosuppressive function of HLA-G in tumorigenesis, the present findings were surprising. Interestingly, similar results were found for other checkpoint molecules, such as PD-1, CTLA-4, TIM-3, and LAG-3 (90). Consistent with the present results, the findings of previous studies showed that high expression levels of HLA-G were related to better OS and DFS in rectal cancer patients but worse OS in pancreatic carcinoma patients (91, 92). Inconsistent with the present results, the findings of previous studies showed that high expression levels of HLA-G were an independent marker of poor prognosis in some tumors, including NSCLC, ovarian, breast, colorectal, hepatocellular and endometrial cancers (93–96). Such conflicting survival results indicate that HLA-G may have different biological roles in different tumor types. Better knowledge of the HLA-G molecule is urgently required and will provide a rationale for novel immunotherapies.

To date, little is known about the relationship between HLA-G expression and tumour-infiltrating immune cells in the tumor microenvironment, which dictate the functional orientation of associated immune checkpoints. To determine why HLA-G was associated with survival, we further investigated the relationships between infiltrating immune cells and HLA-G expression. Our analysis indicated that HLA-G highly expressed in the tumor microenvironment have positive associations with tumour-infiltrating immune cells, including B cells, CD8+ T cells, CD4+ T cells, macrophages, DCs, and neutrophils (**Supplementary Figure 4**). We speculate that HLA-G expression may indirectly recruit immune cells by regulating the secretion of cytokines in the tumor microenvironment. Especially, upregulated expression of HLA-G by tumor cells profoundly affected tumour-specific T cell immunity in the tumor microenvironment. As expected, we found that the functions of HLA-G and its coexpressed genes were primarily related to T cell regulation using GO enrichment analysis. Indeed, HLA-G is thought to be involved in T cell exhaustion and to negatively regulate the immune response in tumors. A recent study focused on tumour-infiltrating CD8+ T cells that express the HLA-G receptor ILT2 in clear cell renal cell carcinoma (ccRCC), and the results emphasize the potential of therapeutically targeting the HLA-G/ILT2 checkpoint in HLA-G+ tumors (7). Another study suggested that ILT2 functions as a negative regulator of human CD8+ effector T cells and that blocking ILT2 represents a unique strategy to enhance BiTE molecule therapeutic activity against solid tumors (97). The development of cancer therapies based on T cell activation must consider such HLA-associated immune evasion mechanisms, as alterations in their expression occur early and frequently in the majority of types of cancer (98). In summary, HLA-G expression were not only associated with immune cell infiltration but also showed tumour-promoting and tumour-inhibiting properties.

In order to explain the intertumoral heterogeneity of HLA-G expression, we investigated the molecular characteristics of HLA-G in different types of tumors. Our analysis suggest that the nucleotide variations, and DNA methylation status of the *HLA-G* gene may explain the differential expression of HLA-G across different tumors (**Figure 5**). The molecular characteristics of HLA-G were related to gene expression regulation, mRNA localization, mRNA stability, and protein post-translational modifications, which can affect HLA-G protein expression. Nucleotide variation in the 5′-upstream regulatory region (5′-URR) have been reported to influence the HLA-G protein expression. Two famous genetic alterations in 3′-untranslated region (3′-UTR), including the 14 bp insertion/deletion mutation (5′-ATTTGTTCATGCCT-3′, rs66554220) and the +3142C/G mutation (rs1063320), are associated with *HLA-G* mRNA stability and impact HLA-G expression (99). DNA methylation and histone acetylation, the best characterized epigenetic modification, plays an essential role in modulating the expression of HLA-G. Previous studies showed lower methylation status of HLA-G DNA promoter was associated with higher degree of HLA-G expression (100, 101). Treatment of HLA-G-negative cells with demethylation agents such as 5-aza-2’-deoxycytidine could restored HLA-G transcription due to direct demethylation of the HLA-G promoter (102). Other environmental factor such as hypoxia, cytokines, hormones and even immunotherapy chemicals and radiation have been acknowledged to be related to the regulation of HLA-G expression. We further sought to characterize the transcription factor (TF) targets of HLA-G and found that HIVEP2, MYCN, CIITA, MYC, and IRF1 may be the key transcription factors in the regulation of HLA-G expression (**Table 1**). However, research on the relationship between HLA-G and these TFs is limited. Moreover, after HLA-G mRNA is translated to a functional protein, it needs undergo various post-translational modifications such as multimerization, glycosylation, and ubiquitination that may influence biological functions of HLA-G. Thus, due to complexity of mechanisms involved in regulation of HLA-G expression, diversity of HLA-G isoforms, and interaction among different types of immune cells that varies in a particular condition, more studies on the implications of HLA-G-mediated immune tolerance in the various types of human tumors are warranted.

The limitations of this study, as following parts: (i) lack of the data on HLA-G protein expression across various types of cancer, might help to clarify and corroborate our findings; (ii) lack of HLA-G inhibition experiments to explain the causal relationship; (iii) lack of the proteomic data between HLA-G and other HLA class I expression, might help to develop of novel immunological therapeutic approaches for cancer.

In conclusion, we believe that HLA-G can serve as a potential prognostic factor as well as therapeutic target in tumors, especially in renal cell carcinoma and thyroid carcinoma. The current findings may provide novel insights for blockade of the HLA-G/ILT axis, which holds potential for the development of more effective antitumor treatments in cancers.

## Data Availability Statement

The original contributions presented in the study are included in the article/**Supplementary Material**. Further inquiries can be directed to the corresponding authors.

## Author Contributions

Study design: H-HX and W-HY. Primary analysis: H-HX and JG. Validation: D-PX and LL. Writing: H-HX. Review editing: W-HY. All authors contributed to the article and approved the submitted version.

## Funding

This work was supported by grants from National Natural Science Foundation of China (81901625), National Natural Science Foundation of Zhejiang province (LY20H100004), Zhejiang Province Public Welfare Technology Application Research Project (2017C33217), and Science and Technology Bureau of Taizhou (1701ky26, 1901ky05).

## Conflict of Interest

The authors declare that the research was conducted in the absence of any commercial or financial relationships that could be construed as a potential conflict of interest.

## Supplementary Material

Supplementary Figure 1 Tissue-restricted expression pattern of HLA-G on human physiological tissues (HPA dataset). HPA, Human Protein Atlas; TPM, transcript per million.

Supplementary Figure 2 The prognostic values of HLA-G expression on relapse free survival in 21 cancer types patients (Kaplan-Meier Plotter). Survival curves for high (red) and low (black) expression groups dichotomized at the optimal cutpoint are plotted. The X-axis represents time and the Y-axis represents survival rate.

Supplementary Figure 3 The prognostic values of HLA-G expression in 33 cancer types patients (GEPIA). **(A)** overall survival (OS), **(B)** disease free survival (DFS). Survival curves for high (red) and low (blue) expression groups dichotomized at the optimal cutpoint are plotted. The X-axis represents time and the Y-axis represents survival rate. 95% confidence intervals for each group are also indicated by dotted lines.

Supplementary Figure 4 The correlation between HLA-G expression and immune cell infiltration (TIMER).

Supplementary Table 2 Co-expression genes of HLA-G molecule in KIRC (cBioPortal).

Supplementary Table 3 The correlation between HLA-G expression and immune cell infiltration (CIBERSORT).

## Glossary

**Table:**

Affy	Affymetrix
ACC	Adrenocortical carcinoma
BLCA	Bladder Urothelial Carcinoma
BRCA	Breast invasive carcinoma
CESC	Cervical squamous cell carcinoma and endocervical adenocarcinoma
CHOL	Cholangiocarcinoma
COAD	Colon adenocarcinoma
DFS	disease free survival
DLBC	Diffuse Large B-cell Lymphoma
CTLA-4	cytotoxic T lymphocyte-associated protein 4
ESCA	Esophageal carcinoma
GBM	Glioblastoma multiforme
GEO	Gene Expression Omnibus
GO	Gene Ontology
GTEx	(Genotype-Tissue Expression)
HLA-G	Human leukocyte antigen G
HNSC	Head and Neck squamous cell carcinoma
KEGG	Kyoto Encyclopedia of Genes and Genomes
KICH	Kidney Chromophobe
KIRC	Kidney renal clear cell carcinoma
KIRP	Kidney renal papillary cell carcinoma
LAML	Acute Myeloid Leukemia
LGG	Brain Lower Grade Glioma
LIHC	Liver hepatocellular carcinoma
LUAD	Lung adenocarcinoma
LUSC	Lung squamous cell carcinoma
MESO	Mesothelioma
OS	overall survival
OV	Ovarian serous cystadenocarcinoma
PAAD	Pancreatic adenocarcinoma
PCPG	Pheochromocytoma and Paraganglioma
PD-1 (PDCD1)	programmed cell death protein 1
PDAC	Pancreatic ductal adenocarcinoma
PRAD	Prostate adenocarcinoma
READ	Rectum adenocarcinoma
RNAseq	RNA sequencing
SARC	Sarcoma
SKCM	Skin Cutaneous Melanoma
STAD	Stomach adenocarcinoma
STES	Stomach and Esophageal carcinoma
TCGA	The Cancer Genome Atlas
TF	transcription factor
TGCT	Testicular Germ Cell Tumors
TPM	transcripts per million
THCA	Thyroid carcinoma
THYM	Thymoma
UCEC	Uterine Corpus Endometrial Carcinoma
UCS	Uterine Carcinosarcoma
UVM	Uveal Melanoma
