# Supplementary figures and images for: Comprehensive Transcriptomic Analysis Reveals the Role of the Immune Checkpoint HLA-G Molecule in Cancers
Source: Front Immunol. 2021 Jul 1;12:614773. doi: 10.3389/fimmu.2021.614773 (PMC8281136; doi:10.3389/fimmu.2021.614773)

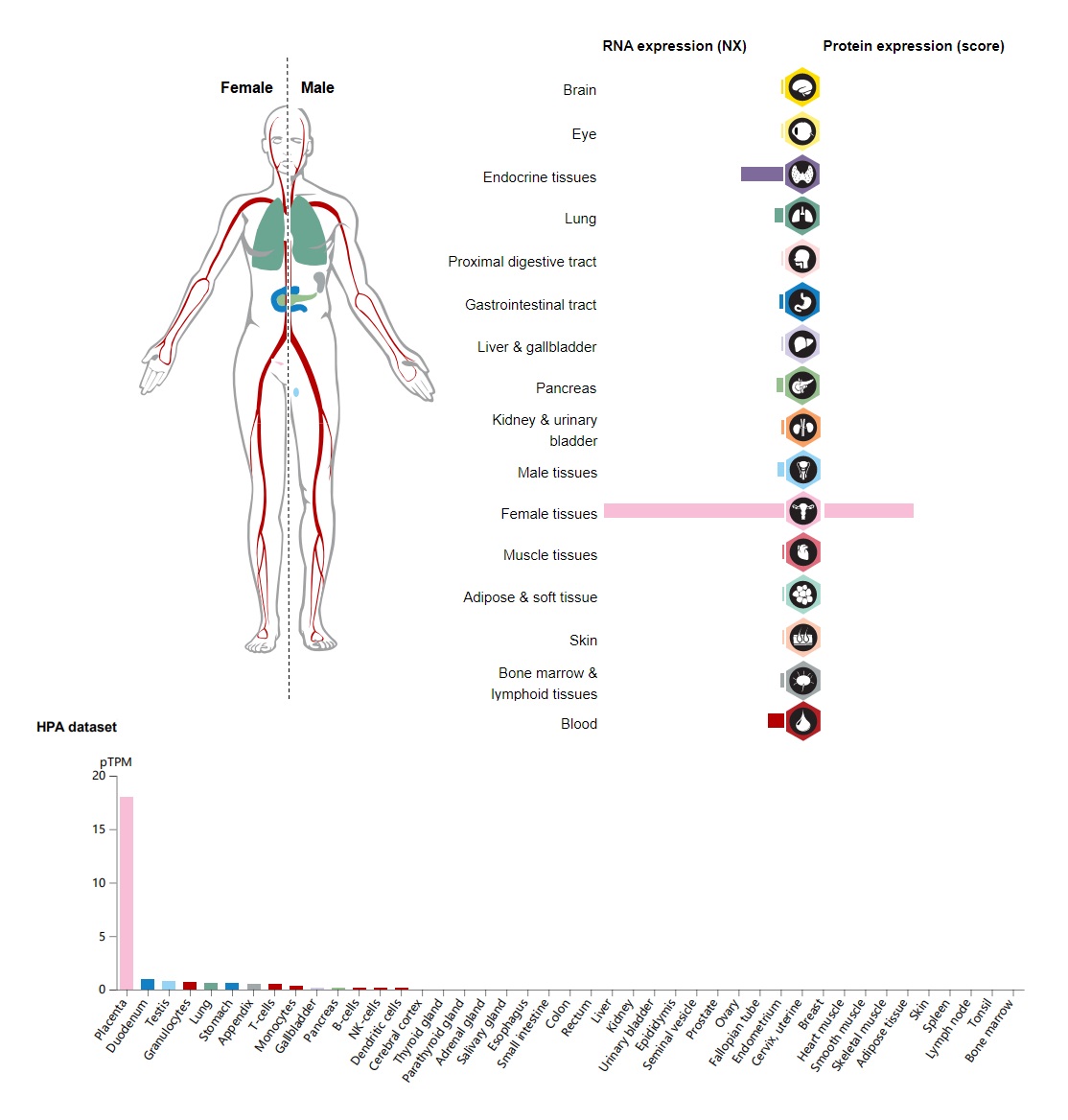

Supplement: Supplementary Figure 1 — Tissue-restricted expression pattern of HLA-G on human physiological tissues (HPA dataset). HPA, Human Protein Atlas; TPM, transcript per million. [file Image_1.jpeg]

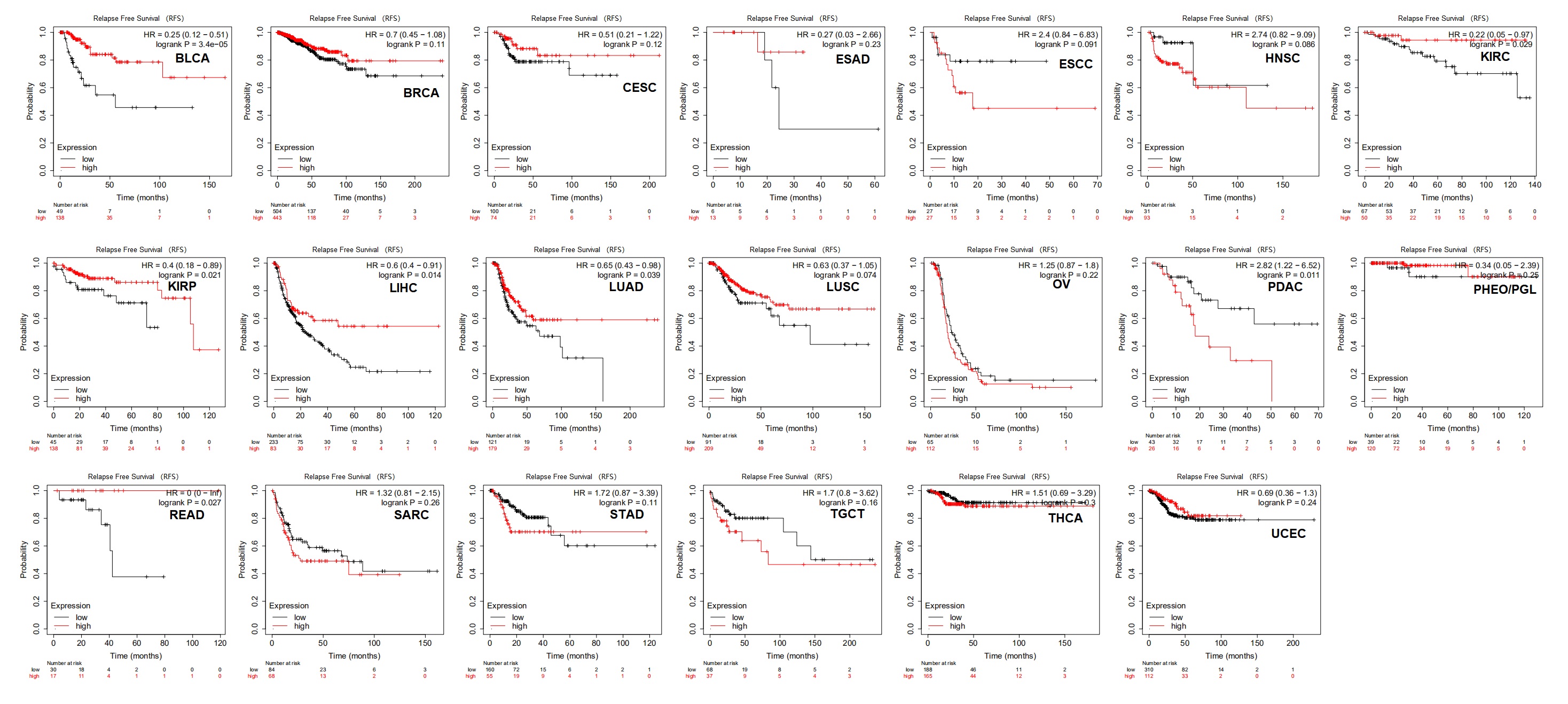

Supplement: Supplementary Figure 2 — The prognostic values of HLA-G expression on relapse free survival in 21 cancer types patients (Kaplan-Meier Plotter). Survival curves for high (red) and low (black) expression groups dichotomized at the optimal cutpoint are plotted. The X-axis represents time and the Y-axis represents survival rate. [file Image_2.jpeg]

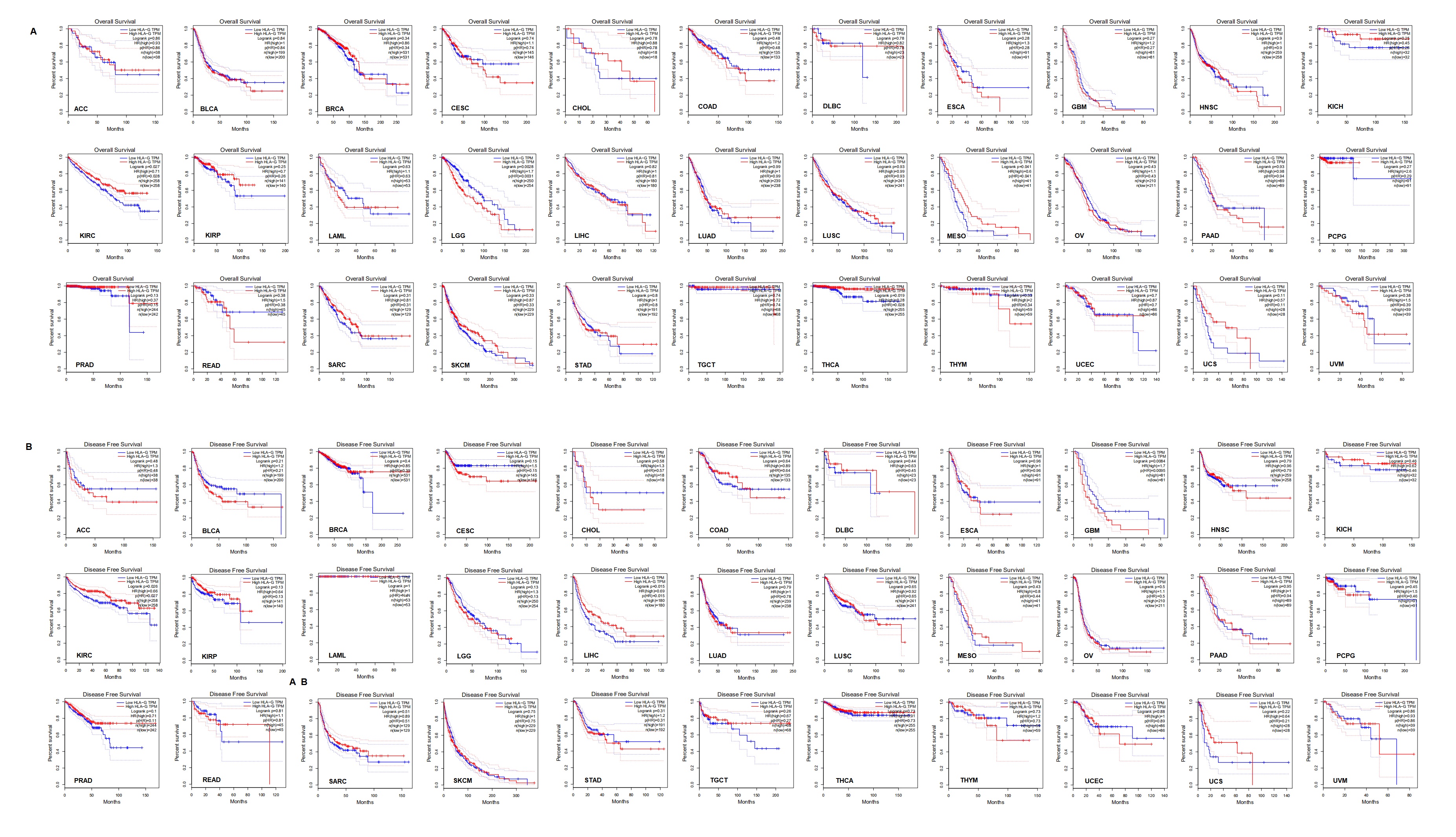

Supplement: Supplementary Figure 3 — The prognostic values of HLA-G expression in 33 cancer types patients (GEPIA). (A) overall survival (OS), (B) disease free survival (DFS). Survival curves for high (red) and low (blue) expression groups dichotomized at the optimal cutpoint are plotted. The X-axis represents time and the Y-axis represents survival rate. 95% confidence intervals for each group are also indicated by dotted lines. [file Image_3.jpeg]

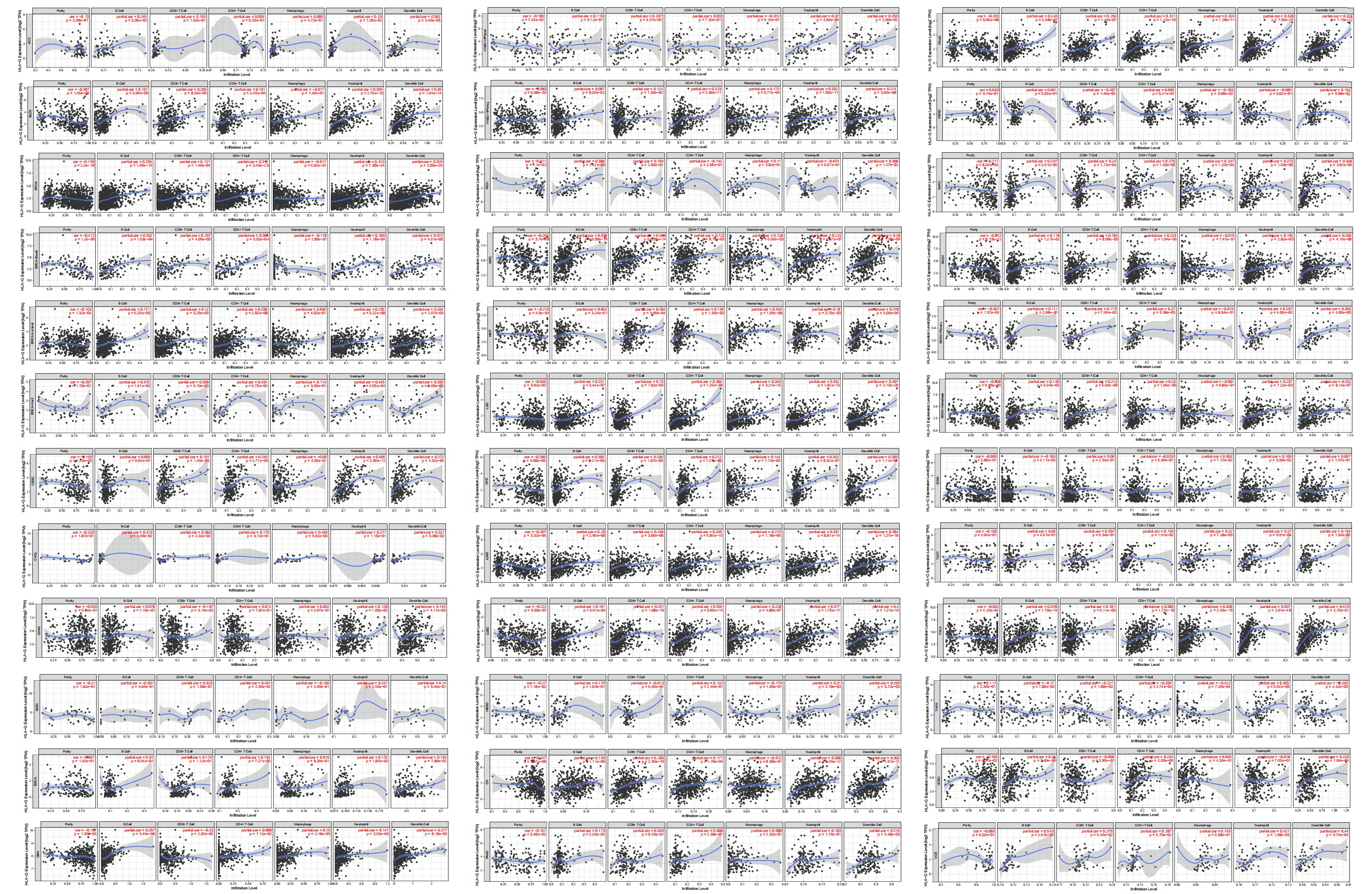

Supplement: Supplementary Figure 4 — The correlation between HLA-G expression and immune cell infiltration (TIMER). [file Image_4.jpeg]
