# Supplementary material for: Comprehensive Transcriptomic Analysis Reveals the Role of the Immune Checkpoint HLA-G Molecule in Cancers
Source: Front Immunol. 2021 Jul 1;12:614773. doi: 10.3389/fimmu.2021.614773 (PMC8281136; doi:10.3389/fimmu.2021.614773)
Supplement: Supplementary file 5 [file Table_1.docx]

| Table S1. The mRNA expression of HLA-G in different types of tumors at transcriptome level (ONCOMINE) | | | | | |
| --- | --- | --- | --- | --- | --- |
| **Cancer** | **Cancer Type** | **Fold change** | ***P*-value** | **t-test** | **Reference** |
| **Brain and CNS Cancer** | Glioblastoma | 6.592 | 8.80E-18 | 18.529 | (17) |
|  | Oligodendroglioma | 2.652 | 6.09E-4 | 7.490 | (17) |
|  | Anaplastic Oligodendroglioma | 2.883 | 0.014 | 5.566 | (17) |
|  | Pilocytic Astrocytoma | 4.367 | 6.84E-5 | 6.806 | (18) |
|  | Glioblastoma | 2.757 | 9.67E-6 | 8.266 | (19) |
|  | Brain Glioblastoma | 3.614 | 2.80E-11 | 23.008 | (19) |
|  | Anaplastic Astrocytoma | 2.443 | 3.90E-11 | 9.311 | (20) |
|  | Glioblastoma | 3.653 | 3.65E-21 | 11.839 | (20) |
|  | Diffuse Astrocytoma | 2.928 | 7.34E-4 | 5.092 | (20) |
|  | Oligodendroglioma | 1.767 | 3.97E-8 | 5.990 | (20) |
|  | Glioblastoma | 2.508 | 4.39E-7 | 8.062 | (21) |
|  | Astrocytoma | 1.799 | 0.001 | 4.245 | (21) |
|  | Oligodendroglioma | 1.854 | 6.84E-4 | 5.441 | (21) |
|  | Glioblastoma | 1.902 | 4.49E-6 | 5.689 | (22) |
|  | Anaplastic Oligodendroglioma | 2.512 | 1.06E-5 | 5.407 | (23) |
|  | Anaplastic Oligoastrocytoma | 2.213 | 0.006 | 3.726 | (23) |
| **Breast Cancer** | Invasive Ductal Breast Carcinoma Stroma | 1.726 | 4.19E-5 | 4.921 | (24) |
|  | Medullary Breast Carcinoma | 2.506 | 1.49E-8 | 7.211 | (25) |
|  | Invasive Lobular Breast Carcinoma | 1.832 | 0.043 | 1.996 | (26) |
| **Cervical Cancer** | Cervical Squamous Cell Carcinoma | 1.641 | 9.16E-9 | 8.306 | (27) |
| **Esophageal Cancer** | Esophageal Adenocarcinoma | 2.204 | 6.36E-5 | 5.236 | (28) |
|  | Esophageal Adenocarcinoma | 2.359 | 0.001 | 3.991 | (29) |
|  | Barrett’s Esophagus | 1.766 | 5.26E-4 | 4.134 | (28) |
|  | Barrett’s Esophagus | 1.907 | 5.21E-5 | 4.440 | (29) |
| **Gastric Cancer** | Gastric Intestinal Type Adenocarcinoma | 1.623 | 5.75E-4 | 3.525 | (30) |
| **Head and Neck Cancer** | Oral Cavity Squamous Cell Carcinoma Epithelia | 2.067 | 6.64E-6 | 5.984 | (31) |
|  | Tongue Squamous Cell Carcinoma | 2.100 | 2.86E-10 | 7.501 | (32) |
|  | Floor of the Mouth Carcinoma | 3.209 | 5.61E-7 | 7.100 | (33) |
|  | Oropharyngeal Carcinoma | 2.508 | 1.15E-5 | 5.476 | (33) |
|  | Tongue Carcinoma | 2.697 | 2.15E-6 | 5.496 | (33) |
|  | Tonsillar Carcinoma | 2.752 | 0.002 | 3.807 | (33) |
|  | Oral Cavity Carcinoma | 3.087 | 0.006 | 3.923 | (33) |
|  | Tongue Squamous Cell Carcinoma | 1.846 | 2.71E-9 | 6.914 | (34) |
|  | Head and Neck Squamous Cell Carcinoma | 2.054 | 0.005 | 3.987 | (35) |
|  | Head and Neck Squamous Cell Carcinoma | 2.295 | 1.14E-6 | 6.271 | (36) |
| **Kidney Cancer** | Renal Wilms Tumor | 1.683 | 1.73E-8 | 8.988 | (37) |
|  | Clear Cell Sarcoma of the Kidney | 2.046 | 3.12E-7 | 8.586 | (37) |
|  | Hereditary Clear Cell Renal Cell Carcinoma | 3.188 | 6.92E-17 | 13.474 | (38) |
|  | Non-Hereditary Clear Cell Renal Cell Carcinoma | 2.338 | 6.09E-12 | 9.757 | (38) |
|  | Clear Cell Renal Cell Carcinoma | 3.298 | 1.62E-8 | 9.304 | (39) |
|  | Clear Cell Renal Cell Carcinoma | 2.729 | 5.15E-9 | 7.372 | (40) |
|  | Clear Cell Renal Cell Carcinoma | 1.986 | 8.52E-5 | 5.453 | (41) |
|  | Clear Cell Renal Cell Carcinoma | 8.072 | 6.44E-5 | 8.243 | (42) |
|  | Papillary Renal Cell Carcinoma | 3.690 | 1.16E-11 | 9.991 | (40) |
|  | Papillary Renal Cell Carcinoma | 4.031 | 7.08E-4 | 5.581 | (42) |
|  | Renal Oncocytoma | 2.751 | 4.21E-9 | 7.645 | (40) |
|  | Renal Oncocytoma | 4,141 | 0.001 | 5.116 | (42) |
| **Leukemia** | Chronic Lymphocytic Leukemia | 1.871 | 0.001 | 3.926 | (43) |
| **Liver Cancer** | Liver Cell Dysplasia | 1.827 | 2.18E-5 | 5.359 | (44) |
|  | Hepatocellular Carcinoma | 1.549 | 9.95E-13 | 9.169 | (45) |
|  | Cirrhosis | 2.278 | 9.32E-7 | 6.640 | (44) |
|  | Cirrhosis | 1.922 | 6.86E-12 | 9.753 | (45) |
| **Lung Cancer** | Squamous Cell Lung Carcinoma | 1.879 | 1.42E-7 | 5.842 | (34) |
| **Lymphoma** | Hodgkin's Lymphoma | 1.562 | 5.16E-5 | 4.711 | (46) |
| **Melanoma** | Benign Melanocytic Skin Nevus | 4.856 | 2.90E-4 | 4.089 | (47) |
|  | Cutaneous Melanoma | 7.974 | 3.31E-5 | 7.563 | (47) |
| **Myeloma** | Smoldering Myeloma | 1.716 | 4.67E-6 | 5.442 | (48) |
| **Pancreatic Cancer** | Pancreatic Adenocarcinoma | 6.739 | 0.002 | 4.416 | (49) |
| **Sarcoma** | Clear Cell Sarcoma of the Kidney | 2.046 | 3.12E-7 | 8.586 | (50) |
| **Other Cancer** | Testicular Intratubular Germ Cell Neoplasia | 2.530 | 0.009 | 5.761 | (51) |
|  | Testicular Embryonal Carcinoma | 8.262 | 0.003 | 4.780 | (51) |
|  | Testicular Yolk Sac Tumor | 2.343 | 0.006 | 4.049 | (51) |
|  | Testicular Teratoma | 4.308 | 0.019 | 3.168 | (51) |
|  | Familial Parathyroid Hyperplasia | 2.342 | 0.004 | 4.006 | (52) |
|  | Non-Familial Multiple Gland Neoplasia | 1.754 | 0.001 | 3.898 | (52) |
|  | Actinic (Solar) Keratosis | 1.619 | 0.009 | 2.938 | (53) |
|  | Embryonal Carcinoma | 3.414 | 5.01E-8 | 9.235 | (54) |
|  | Teratoma, NOS | 3.149 | 2.06E-7 | 9.551 | (54) |
|  | Seminoma, NOS | 3.016 | 3.50E-7 | 8.429 | (54) |
